# Hospital Meetings

**Published:** 1901-08-03

**Authors:** 


### HOSPITAL MEETINGS.

### EAST LONDON HOSPITAL FOR CHILDREN.

OWING to the severe thunderstorm of Thursday afternoon (25th ult.) there was only a small attendance of Governors at the half-yearly court, and no meeting could be held as the number did not form a quorum. The following are the chief details of the report: The receipts for the six months ended June 30th, 1901, amounted to £4,266 5s. 11d., of which

£977 2s. 3d. were in annual subscriptions, £2,107 6s. 3d. in donations, and £561 6s. 8d. in legacies, the remainder being miscellaneous receipts and dividends on stock. The general result, the report states, is hardly satisfactory; 724 in-patients were admitted during the year, 14,747 out-patients, and 2,230 casualty patients. At the convalescent home there were on January 1st 18 patients; 153 have been sent down during the past six months. The Board earnestly appeal for means to enable them to increase the accommodation of the hospital. The new buildings referred to in the report for 1900 have not yet been begun, but the plans for the erection of the new mortuary, etc., have been settled, and tenders will shortly be invited. The Board trust that further donations may be at once forthcoming, to enable them to include the new laundry in the tenders, and to proceed with some of the other buildings which are badly wanted. At present only £681 19s. has been collected in addition to the £500 granted towards the mortuary by the Prince of Wales's Hospital Fund for London. A festival dinner was held in April under the presidency of the Lord Mayor, whose appeal resulted in contributions amounting to over £1,960, including annual subscriptions, and £576 18s. in donations to the New Buildings Fund. The Board desire to take this opportunity of thanking his Lordship for the very active part he so kindly took in connection with this festival. The results of a concert at the Albert Hall on June 8th amounted to £208.

